# Integrated transcriptomic and metabolomic analyses elucidate the regulatory role of SlBEL11 in tomato fruit ripening

**DOI:** 10.3389/fpls.2025.1666515

**Published:** 2025-09-02

**Authors:** Xiufen Dong, Jie Lu, Yun Guo, Qihan Zhang, Qin Yang, Jingyi Peng, Li Tian

**Affiliations:** ^1^ Collaborative Innovation Center for Efficient and Green Production of Agriculture in Mountainous Areas of Zhejiang Province, College of Horticulture Science, Zhejiang Agriculture and Forestry (A&F) University, Hangzhou, China; ^2^ Key Laboratory for Quality and Safety Control of Subtropical Fruits and Vegetables, Collaborative Innovation Center for Efficient and Green Production of Agriculture in Mountainous Areas of Zhejiang Province, Ministry of Agriculture and Rural Affairs, College of Horticulture Science, Zhejiang Agriculture and Forestry (A&F) University, Hangzhou, China

**Keywords:** tomato, SlBEl11, metabolomics, transcriptomics, fruit ripening

## Abstract

Transcription factors serve as key regulators in orchestrating fruit ripening, modulating gene expression networks that govern physiological processes such as color change, texture softening, and sugar accumulation in response to hormonal signals like ethylene and abscisic acid. SlBEL11, a BEL1-like transcription factor, was previously shown to mediate premature fruit abscission in tomato. However, the molecular mechanisms by which SlBEL11 regulates ripening, including its direct target genes, metabolic pathways, and interaction networks, remain largely unknown. In this study, an integrated approach combining untargeted metabolomics and transcriptomics was employed to investigate the metabolic and molecular alterations in wild-type (WT) and *SlBEL11*-RNAi knockdown tomato fruits. UPLC-MS/MS analysis identified a total of 189 differentially expressed metabolites (DEMs), with 74 upregulated and 115 downregulated in *SlBEL11*-RNAi compared to the WT. Meanwhile, transcriptome analysis uncovered 665 differentially expressed genes (DEGs), including key regulators directly associated with ripening processes. Conjoint analysis demonstrated significant enrichment of both DEGs and DEMs in critical metabolic pathways, such as ascorbate and aldarate metabolism, glycolysis, and phenylpropanoid biosynthesis. These pathways were demonstrated to be directly or indirectly modulated by SlBEL11, highlighting its central role in coordinating metabolic reprogramming during fruit maturation. Specifically, SlBEL11 appears to fine-tune the balance among energy supply, cell wall modification, and antioxidant biosynthesis, thereby influencing fruit texture, nutritional quality, and shelf-life. Collectively, these findings not only provide novel insights into the regulatory network of SlBEL11 in tomato ripening but also offer potential genetic targets for the development of tomato cultivars with improved postharvest traits and enhanced fruit quality and secondary metabolite production.

## 1 Introduction

Tomato is a globally important economic crop and a model species for fleshy fruit development research. Its ripening process directly influences the nutritional quality, storage processing, and commercial value of the harvested fruits. Fruit firmness, a central phenotypic trait, is regulated by multiple metabolic pathways. Specifically, ascorbic acid metabolism impacts cell wall cross-linking through hydroxyproline synthesis (Vaughan, 1973), henylpropanoid-mediated lignin deposition directly enhances cell wall mechanical strength (Liu et al., 2016), and energy supply from glycolysis may indirectly modulate the softening rate by regulating cell wall degrading enzyme activities (Adetunjia et al., 2016). Concurrently, tomato ripening entails a cascade of physiological and biochemical transitions, such as chlorophyll degradation, carotenoid biosynthesis and volatile compound accumulation (Ming et al., 2023; Gambhir et al., 2024), under tight regulation of complex transcriptional networks and phytohormone signaling pathways, notably ethylene and abscisic acid.

Previous studies have uncovered the pivotal roles of several transcription factor (TF) families during tomato fruit ripening. For instance, tomato MADS-RIN protein regulates fruit ripening through direct binding to CArG box element in the promoter regions of ripening-associated genes and forming multi-complexes with other MADS-box proteins like FUL1 and FUL2 (Wang et al., 2014). NAC family protein NOR-like1 positively regulates the expression of ethylene biosynthesis related genes (*SlACS2*, *SlACS4*), color formation (*SlGgpps2*, *SlSGR1*), and cell wall metabolism (*SlPG2a*, *SlPL*, *SlCEL2*, *SlEXP1*) to promote ripening initiation (Gao et al., 2018). Ethylene responsive factor SlERF6 exhibits tissue-specific regulatory patterns and positively regulates tomato fruit ripening through modulating the expression of another transcription factors, SlDEAR2 and SlTCP12 (Chen et al., 2025).

BEL1-like (BELL) proteins are ubiquitous transcription factors in plants. They belong to three-amino acid-loop-extension (TALE) superfamily, and usually form heterodimers with other proteins to regulate organogenesis, hormone metabolism, and environmental adaptability (Sharma et al., 2014; Wang et al., 2025). For instance, in *Arabidopsis thaliana*, members of the BEL1-like homeodomain family, including PENNYWISE (PNY), POUND-FOOLISH (PNF), ARABIDOPSIS THALIANA HOMEOBOX 1 (ATH1), and VAAMANA (VAN), interact with KNOX family proteins BREVIPEDICELLUS (BP) and SHOOT MERISTEMLESS (STM) through heterodimer formation. This regulatory complex orchestrates critical developmental processes, such as apical meristem maintenance, inflorescence architecture specification, and floral transition (Smith and Hake, 2003; Bhatt et al., 2004; Kanrar et al., 2006; Rutjens et al., 2009). In potato (*Solanum tuberosum* L.), StBEL5 interacts with potato homeobox 1 (POTH1) and modulates tuber formation by suppressing the expression of a gibberellin biosynthesis gene *GA20ox1* (Chen et al., 2004). In tomato, fourteen BEL1-like genes have been identified (He et al., 2022b). Among them, two members have been reported to be involved in fruit development. SlBL4 acts as a central regulator coordinating chlorophyll homeostasis by modulating chloroplast ultrastructure formation, pectin methylesterase-mediated cell wall remodeling, and carotenoid biosynthesis during fruit maturation. Meanwhile it drives the expansion of pedicel abscission zone via auxin gradient redistribution and programmed cell death, thereby mediating ripening-associated fruit detachment (Yan et al., 2020, 2021). In contrast, SlBEL11 is hypothesized to be a downstream regulator of ethylene signaling during ripening, which is supported by its marked upregulation during the breaker-stage and the presence of ethylene-responsive elements (EREs) in its promoter (He et al., 2022b). Previous studies revealed that silencing *SlBEL11* prevents premature fruit drop, affects chloroplast development and enhances chlorophyll accumulation in tomato fruit (Meng et al., 2018; Dong et al., 2024). However, the molecular mechanisms through which SlBEL11 regulates fruit ripening, including its direct target genes, metabolic pathways, and epigenetic mechanisms, remains unclear. The breaker stage, characterized by the initiation of chlorophyll degradation and the onset of carotenoid accumulation (as evidenced by the first visible color transition from green to yellowish-orange at the stylar end), represents a phenologically critical checkpoint in tomato fruit ripening (Sato et al., 2012) This phase coincides with the burst of ethylene biosynthesis and transcriptional activation of ripening-related genes governing cell wall modification, volatile synthesis, and chloroplast-to-chromoplast transition (Klee and Giovannoni, 2011; Gambhir et al., 2024). Selection of this developmental window is grounded in its role as a definitive molecular switch from maturation to ripening—a period when transcriptional reprogramming events directly associated with quality trait establishment are initiated. Furthermore, *SlBEL11* exhibits stage-specific upregulation during this phase, as previously reported (He et al., 2022a), making it an optimal time point to dissect its regulatory hierarchy. Sampling at this stage minimizes confounding effects from pre-ripening developmental processes while capturing early transcriptomic and metabolomic signatures linked to ripening progression, thereby enabling precise identification of SlBEL11-dependent pathways before secondary regulatory networks mask primary molecular responses.

Transcriptomics and metabobolics are the main approaches that utilize high-throughput sequencing technologies. Transcriptomics, leveraging high-throughput sequencing technologies (e.g., Illumina platforms), enable deep sequencing and differential expression analysis of whole transcriptomes to dissect molecular mechanisms at the gene expression level (Sarfraz et al., 2025). Metabolomics focuses on systematically identifying the composition and dynamics of metabolites in biological samples through high-resolution mass spectrometry, enabling precise quantification to reveal terminal phenotypic responses and biochemical regulatory networks under environmental stress (Oh et al., 2023). This study integrates transcriptomic and metabolomic approches to elucidate the specific regulatory role of the SlBEL11 in tomato fruit ripening. By comparing two groups, a wild-type control with normal *SlBEL11* expression and another with perturbed *SlBEL11* expression, we aim to unravel the precise regulatory mechanisms of SlBEL11 during ripening, thereby providing genetic resources and technical foundations for optimizing secondary metabolite production in tomato.

## 2 Materials and methods

### 2.1 Preparation of plant samples

*SlBEL11*-RNAi transgenic line was kindly donated by Dr. Daqi Fu, School of Food Science and Nutrition Engineering, China Agricultural University. All tomato plants, wild type (Micro-tom) and SlBEL11-RNAi line used in this experiment were cultivated in a growth incubator under photo-cycle condition of 16-h light (22000 Lux) at 25°C and 8-h dark at 20°C and a maintained humidity at 70%~80%. Fresh fruit samples were collected at breaker stage and used for the subsequent transcriptomic and metabolic analyses. Three biological and technical replicates were implemented for both transcriptome and metabolome profiling.

### 2.2 Measurement of tomato fruit firmness

Fruit firmness was measured using a pointer-type fruit firmness tester (Model GY-3, Aipu Measuring Instruments Co., Ltd., China). The test sample was placed face up on a horizontal experimental bench, and the compression force required to break the fruit was recorded. The value was divided by the surface area of the compressed region, and the pressure required per unit area was taken as the firmness metric of the tomato fruit.

### 2.3 Transcriptomics analysis

Tomato fruit samples were flash-frozen in liquid nitrogen, freeze-dried using a vacuum freeze-dryer (Scientz-100F), and ground into powder with zirconium oxide beads using a mixer mill at 65 Hz for 1 minute. Total RNA was extracted using a RNA extraction kit (Tiangen Biotech, Beijing, China) according to the manufacturer’s instructions. RNA quantity and purity were measured using a Nano Drop ND-1000 (Thermo Fisher), with acceptable thresholds set as A260/A280 = 1.8-2.1 and A260/A230 ≥ 2.0. RNA integrity was evaluated using an Agilent Bioanalyzer 2100, and only samples with RNA Integrity Number (RIN) ≥ 7.0 were selected for downstream analysis. cDNA libraries were constructed using the Illumina TruSeq Stranded mRNA Library Prep Kit, including mRNA enrichment, fragmentation, double-stranded cDNA synthesis, end repair, adapter ligation, and PCR amplification. After quality validation, libraries were sequenced on an Illumina NovaSeq 6000 system (LC-Bio, Hangzhou, China) in paired-end (PE150) mode, generating ≥6 GB of raw data per sample.

Raw sequencing reads were preprocessed using Fastp to remove low-quality reads (Q < 20), adapter-contaminated sequences, and reads with >5% ambiguous bases (N). Paired-end reads were aligned to the tomato reference genome (SL4.0, downloaded from Sol Genomics Network) using HISAT2 v2.2.1 with parameters: –rna-strandness RF –dta –phred33. Index files were generated using hisat2-build with default settings. Gene expression levels were quantified as Fragments Per Kilobase of transcript per Million mapped reads (FPKM), a widely used metric for estimating transcript abundance. Differential expression analysis was performed using DESeq2 (v1.38.3), with significance thresholds set as |log_2_(fold change)| ≥1 and Benjamini-Hochberg adjusted P-value (FDR) < 0.05. Functional enrichment analysis included KEGG pathway analysis via hypergeometric testing (FDR < 0.05) and Gene Ontology (GO) term analysis using Fisher’s exact test, both referenced against the tomato genome annotation database.

### 2.4 Metabolomics analysis

The pretreatment process for tomato samples was consistent with transcriptomics protocols. A 50 mg aliquot of the powdered sample was mixed with 1 mL of pre-chilled extraction solvent (methanol/water/formic acid, 15:4:1, v/v/v), vortexed, and sonicated in an ice bath (20 kHz, 5-second intervals, total duration 1 hour). The mixture was centrifuged at 8,000 × g for 5 minutes at 4°C, and the supernatant was collected, vacuum-dried, and reconstituted in 80% methanol. After purification via centrifugation (20,000 × g, 20 minutes, 4°C), the solution was filtered through a 0.22 μm cellulose acetate membrane and stored in HPLC vials at -80°C. Three biological replicates were included per group, with quality control (QC) samples prepared by pooling equal amounts of WT and SlBEL11-RNAi extracts. Three consecutive injections of QC samples were performed prior to formal analysis to stabilize the instrument. Chromatographic separation was carried out on an Agilent SB-C18 column (1.8 μm × 2.1 mm × 100 mm) using a UPLC system (ExionLC™ AD) coupled with a 6500 QTRAP mass spectrometer. The mobile phases consisted of 0.1% formic acid in water (A) and 0.1% formic acid in acetonitrile (B), with a gradient program: 95% A to 95% B over 9 minutes, held for 1 minute, then returned to initial conditions in 70 seconds (flow rate: 0.35 mL/min; column temperature: 40°C). Mass spectrometry parameters included electrospray ionization (ESI) in positive/negative switching mode, ion source temperature of 550°C, and spray voltages of ±5,500/4,500 V.

Raw data were processed using MS-DIAL for peak alignment, retention time correction, and peak area extraction. Metabolites were identified by matching accurate mass (mass tolerance < 0.01 Da) and MS/MS spectra (mass tolerance < 0.02 Da) against in-house standards, the Human Metabolome Database (HMDB), and MassBank. Features detected in > 50% non-zero measurements within at least one experimental group were retained for downstream analysis. Differential metabolites were identified through a dual-filter approach combining Orthogonal Partial Least Squares-Discriminant Analysis (OPLS-DA) parameters and statistical criteria: (1) variable importance in projection (VIP) scores > 1,(2) absolute fold-change (FC) ≥ 2 with p< 0.05.

### 2.5 RNA extraction and RT-qPCR analysis

Total RNA was extracted from tomato tissues using the RNApure Plant Kit (CWBIO, Beijing, China). For first-strand cDNA synthesis, 2 μg of total RNA was reverse-transcribed using reverse transcriptase and oligo(dT) primers. Quantitative PCR (qPCR) was performed on a qTOWER3/G real-time system (Analytik Jena, Germany). Each reaction (20 μL total volume) contained 25 ng cDNA, 200 nM of each primer, and 4 μL SuperReal PreMix Plus (Tiangen Biotech, Beijing, China; containing DNA polymerase, dNTPs, and optimized buffer components). The thermal cycling program included an initial denaturation at 95°C for 30 s, followed by 40 cycles of 95°C for 5 s (denaturation) and 59°C for 30 s (annealing/extension). Melt curve analysis was performed to verify amplification specificity. Gene expression levels were normalized to the tomato actin gene as an internal control. the 2^-ΔΔCt^ was rigorously applied for relative quantification of gene expression (Livak and Schmittgen, 2001). The primer sequences used in this study are provided in **Supplementary Table S1**.

### 2.6 Statistical analysis

Data are presented as mean ± standard deviation (SD). Multivariate data analysis and graphical visualization were performed using R (version 4.0.3) and associated R packages.

## 3 Result

### 3.1 Transcriptomic analysis of SlBEL11’s role in tomato fruit ripening

**Figure 1:**
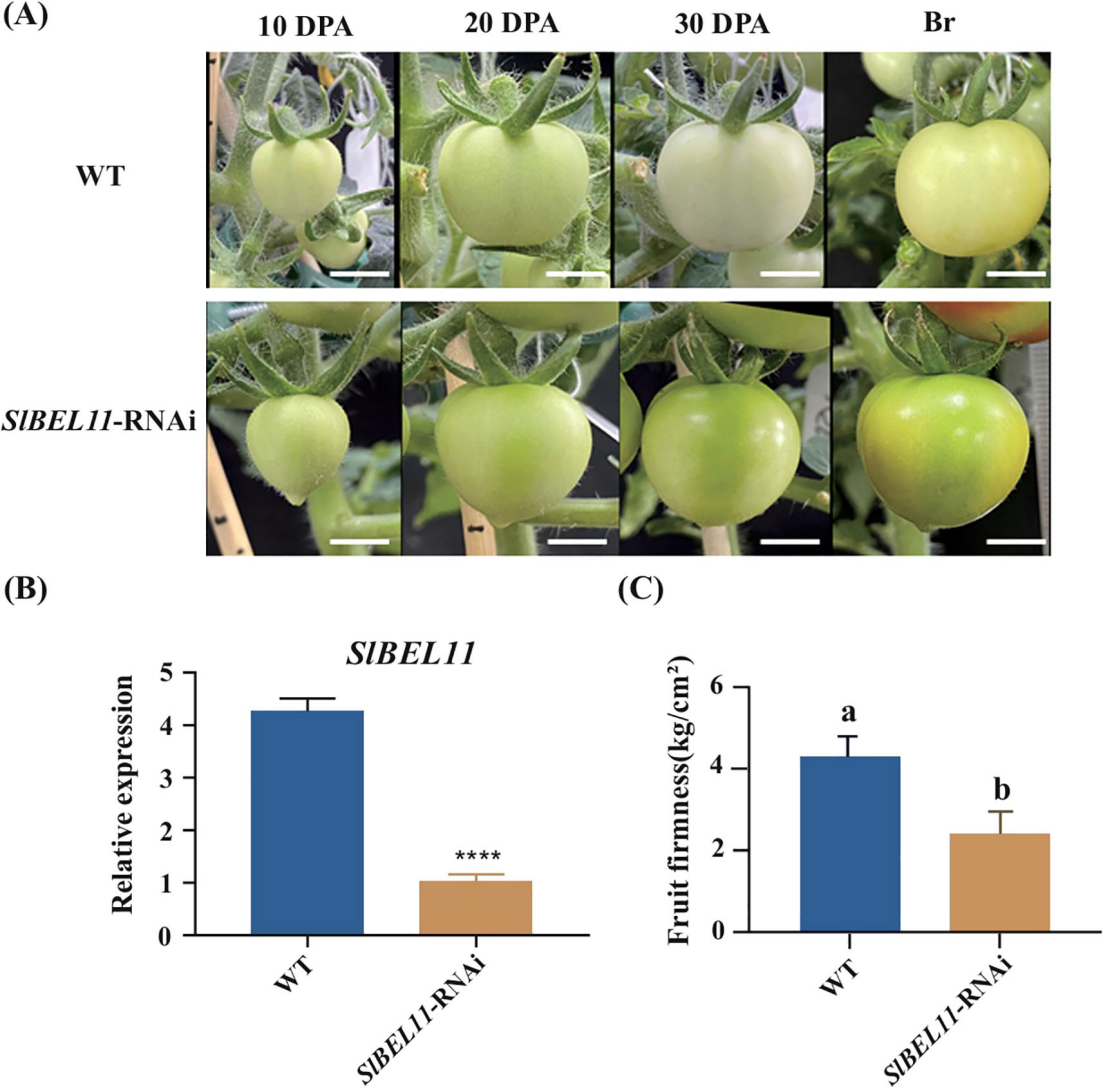
Fruit developmental status and firmness in wild-type and *SlBEL11*-RNAi plants. **(A)** Fruit development stages of WT and *SlBEL11*-RNAi plants, DPA, day post anthesis, Br, breaker, scale=1cm. **(B)** The relative expression of SlBEL11 in WT and *SlBEL11*-RNAi fruits at breaker stage, p < 0.0001. **(C)** The fruit firmness of WT and *SlBEL11*-RNAi fruits at breaker stage. Statistical significance was assessed using a one‐way analysis of variance (ANOVA) with Tukey's multiple comparisons test; different lowercase letters indicate significant differences (P < 0.05). Statistical significance was assessed using a two way analysis of variance (ANOVA)with Sidak's multiple comparisons test. ****P < 0.0001.

Observations of developing fruits in wild-type and *SlBEL11*-RNAi lines revealed that silencing *SlBEL11* expression significantly enhanced chlorophyll accumulation in immature fruits (a phenotype previously reported by Meng et al., 2018). No obvious signs of fruit softening were detected during the growth phase (**Figure 1A**). However, fruits began to abscise progressively upon entering the ripening stages (Dong et al., 2024), with noticeable softening observed via tactile evaluation. Subsequent analysis confirmed the silencing efficiency of *SlBEL11* in transgenic lines, demonstrating a marked reduction in *SlBEL11* transcript levels at the breaker stage fruits (**Figure 1B**). Firmness measurements revealed a 30% reduction in *SlBEL11*-RNAi fruits at breaker stage (**Figure 1C**).

To elucidate the molecular mechanisms, we conducted comparative transcriptome profiling of wild-type and *SlBEL11*-RNAi fruits using Illumina NovaSeq 6000 sequencing. As shown in **Supplementary Table S2**, a total of 13.43 GB of raw data (267,776,280 paired-ended reads) were generated. Stringent quality control using Fastp v0.23.4 was conducted to remove low-quality reads, adapter sequences and reads containing > 5% ambiguous bases (N), yielding 39.72 GB of high-quality clean data (263,556,950 valid reads) with Q30 > 95.97%, and GC content of 42%-45%.

The biological repeatability of the samples was evaluated using Pearson correlation coefficient (**Supplementary Figure S1A**). Intra-group sample correlations exceed R² > 0.9, revealing the reliability and reproducibility of the experimental design. Gene expression levels were normalized using the FPKM method and visualized via violin plots (**Supplementary Figure S1B**) and density distribution map (**Supplementary Figure S1C**). These analyses revealed similar gene expression patterns between groups, with log_10_(FPKM) values concentrated in the range of -2 to 2, indicating that *SlBEL11* silencing did not induce global transcriptional alterations.

### 3.2 GO and KEGG pathway analyses of differentially expressed genes

**Figure 2:**
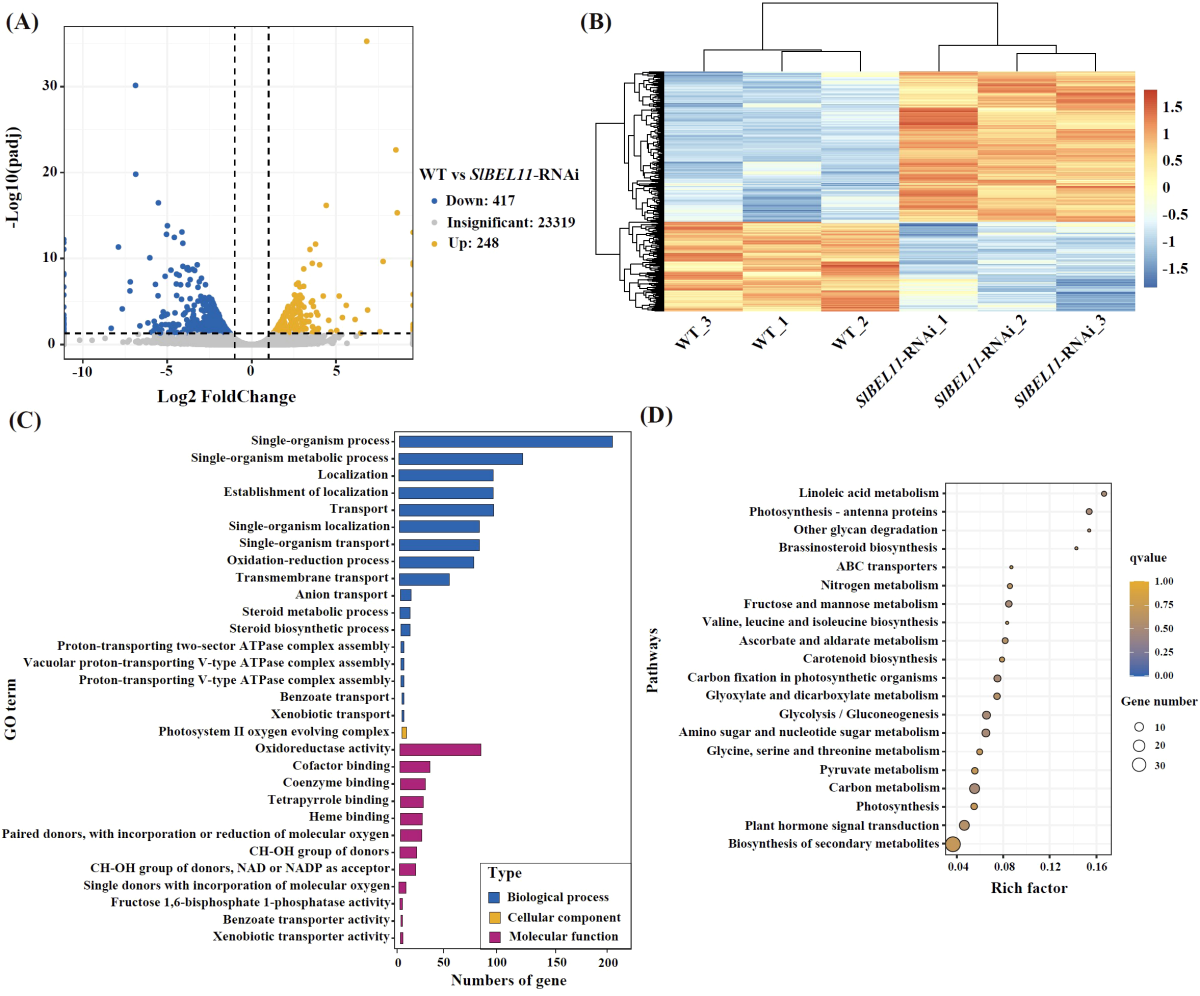
Identification of DEGs in *SlBEL11*-RNAi tomatoes compared to WT group. **(A)** Volcano plot to show the DEGs. **(B)** Cluster heatmap of DEGs. **(C)** GO enrichment analysis of DEGs. **(D)** KEGG enrichment analysis of DEGs.

Differentially expressed genes (DEGs) were further detected using DESeq2 v1.38.3 with a threshold of |log_2_ Fold Change| > 1 and FDR-corrected P < 0.05 (**Figure 2A**, **Supplementary Table S3**). Only 665 DEGs were identified, including 417 up-regulated and 248 down-regulated genes. Hierarchical clustering heatmap (**Figure 2B**) revealed distinct intergroup segregation and tight intragroup clustering of DEGs. To verify the transcriptomic results, 14 DEGs were selected for RT-qPCR analysis (**Supplementary Figure S2**). The expression patterns of the tested DEGs were consistent with that in the transcriptome.

GO enrichment analysis of DEGs are shown in **Figure 2C** and **Supplementary Table S4**. In the category of Biological Process, DEGs are significantly enriched in the pathways of single-organism process like single-organism metabolic process, single-organism localization, single-organism transport, suggesting that SlBEL11 regulates basal physiological functions. The enrichment of DEGs in other processes, such as oxidative-reduction process, localization and transport related processes, are also detected. In the category of molecular function, the significant enrichment of oxidoreductase activity, cofactor binding, and coenzyme binding, further supported the alteration of oxidative-reduction process in *SlBEL11*-RNAi tomatoes. The detection of binding and transport activities, such as tetrapyrrole binding, heme binding, fructose 1,6-bisphosphate 1-phosphatase activity, benzoate and xenobiotic transporters, hinted at potential changes in secondary metabolism. In the category of cellular component, however, only the “photosystem II oxygen evolving complex” was significantly enriched, indicating a potential impact on chloroplast function. This finding aligns with the result of KEGG enrichment analysis (**Figure 2D**, **Supplementary Table S5**) where DEGs clustered in photosynthesis-antenna protein pathways. Additionally, enrichment in linoleic acid metabolism, brassinosteroid biosynthesis and ABC transporter were also detected.

### 3.3 Metabolite statistics and quality control

As the chromatography system/mass spectrometer is in direct contact with the samples, the accumulation of residues in the chromatographic column and the mass spectrometry ion source may cause signal drift or system errors with increasing sample load (Hao et al., 2023). To ensure data reliability and repeatability, three quality control (QC) samples were used for continuous monitoring of the instrument in this study. The superimposed analysis of total ion chromatograms in both positive and negative ion modes showed that the peak intensities and time reproducibility of the QC samples were highly consistent (**Supplementary Figure S3**), demonstrating excellent signal stability of the instrument. Further pearson correlation analysis of the QC samples showed that the correlation coefficients were greater than 0.9 (**Supplementary Figure S4**), confirming the stability of the experimental procedure and the optimal performance of the instrument.

Metabolites were structurally identified by matching retention time, molecular mass (mass error <10 ppm), MS/MS fragmentation patterns, and collision energy against both in-house and public databases. All identifications were subjected to rigorous manual verification. Metabolites with a coefficient of variation (CV) <30% in QC samples were retained for subsequent analysis. A total of 714 metabolites were identified in wild-type (WT) and *SlBEL11*-RNAi tomato samples, spanning 22 metabolic categories, including alcohols(16), alkaloids(41), amino acid and derivatives(92), anthocyanins(12), carbohydrates(20), flavanone(21), flavone(51), flavonoid(18), flavonol(29), indole derivatives(6), isoflavone(5), lipids(75), nucleotide and derivates(59), organic acids and derivatives(106), phenolamides(27), phenylpropanoids(62), polyphenol(7), proanthocyanidins(1), quinones(2), sterides(5), Terpene(13), Vitamins and derivatives(16) and unclassified compounds(30) (**Supplementary Table S6**).

### 3.4 Multivariate statistical analysis of tomato fruits metabolites

**Figure 3:**
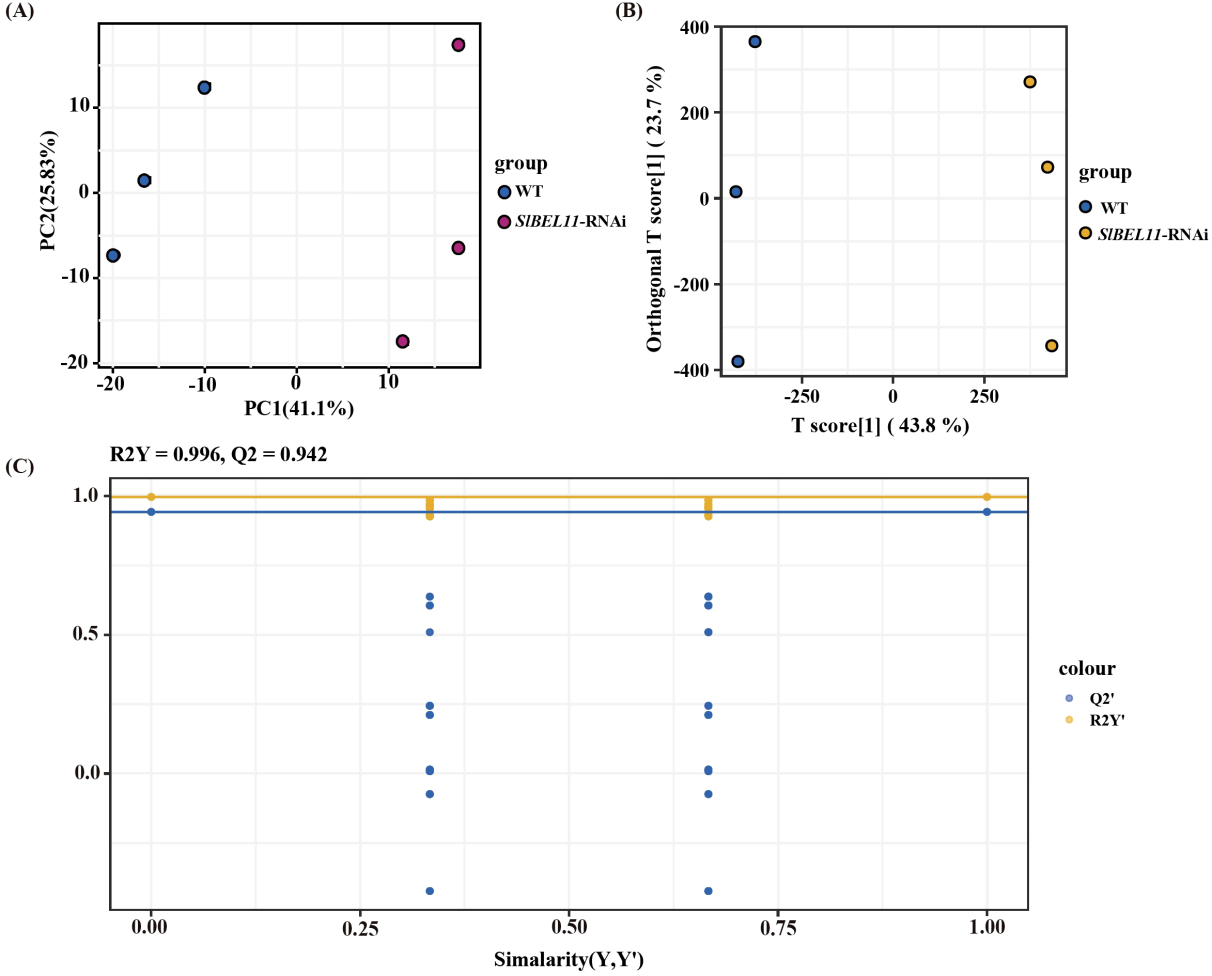
Multivariate statistical analysis of tomato metabolites. **(A)** PCA score plot. **(B)** OPLS-DA score plot. **(C)** 200 permutation tests of the OPLS-DA model verification.

Multivariate analyses of 714 metabolites revealed distinct metabolic profiles between WT and *SlBEL11*-RNAi tomato lines. Principal component analysis (PCA) separated the two groups along the primary axis (PC1, 66.93% variance), with WT and *SlBEL11*-RNAi samples clustering negatively and positively, respectively (**Figure 3A**). While PCA validated experimental stability and intergroup variability, its unsupervised nature limited sensitivity to subtle biological differences. To address this, supervised orthogonal partial least squares-discriminant analysis (OPLS-DA) was employed, yielding an enhanced group discrimination (**Figure 3B**). The model exhibited high reliability (permutation test: R²Y > 0.5, Q² >0.5) with no overfitting (**Figure 3C**), confirming robust metabolic distinctions between genotypes.

### 3.5 Identification and cluster analysis of differential metabolites

**Figure 4:**
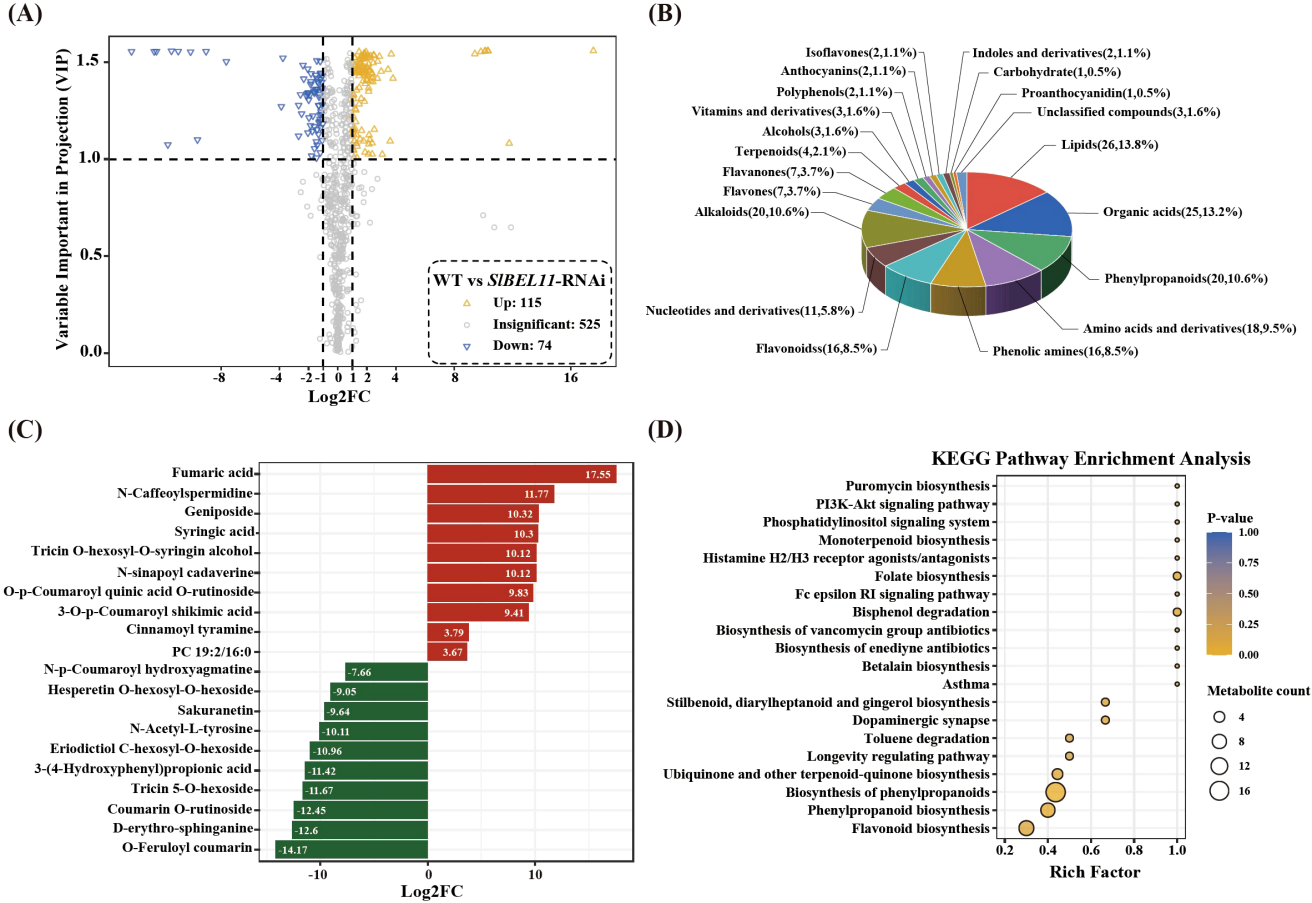
Screening and analysis of differential metabolites in tomato plants of the WT and *SlBEL11*-RNAi groups. **(A)** Volcano plot of differential metabolites. **(B)** Pie Chart of differential metabolite categories. **(C)** Bar plot of the top 10 differentially expressed metabolites based on absolute log_2_ fold change (log_2_FC) values. **(D)** KEGG enrichment analysis of differential metabolites.

A three-tiered screening strategy (absolute FC > 2, P < 0.05, OPLS-DA-derived VIP > 1) was implemented to identify metabolically significant features. A total of 189 differential metabolites were identified in the WT and *SlBEL11*-RNAi tomato samples. As shown in **Figure 4A**, compared with WT, 115 metabolites were up-regulated and 74 were down-regulated in *SlBEL11*-RNAi tomatoes compared to WT. These differential metabolites include 26 lipids, 25 organic acids and derivatives, 20 phenylpropanoids, 18 amino acids and derivatives, 16 phenolic amines, 16 flavonoids, 11 nucleotides and derivatives, 10 flavonols, 10 alkaloids, 7 flavones, 7 flavanones, 4 terpenoids, 3 alcohols, 3 vitamins and derivatives, 2 polyphenols, 2 anthocyanins, 2 isoflavones, 2 indoles and derivatives, 1 carbohydrate, 1 proanthocyanidin and 3 unclassified compounds (**Figure 4B**).

A clustering heatmap was generated to visualize sample relationships and the differences of metabolite intensity, based on the normalized expression values of differential metabolites. As shown in **Supplementary Figure S5**, a distinct hierarchical clustering of metabolite among groups was observed. The top 10 up-regulated and down-regulated differential metabolites were selected using fold change as a criterion. As shown in **Figure 4C**, the top 10 up-regulated differential metabolites included fumaric acid, N-caffeoyl spermidine, geniposide, syringic acid, tricin O-hexosyl-O-syringin alcohol, N-sinapoyl cadaverine, O-p-coumaroyl quinic acid O-rutinoside derivative, 3-O-p-coumaroyl shikimic acid, cinnamoyl tyramine, phosphatidylcholine acyl 19:2/16:0. The top 10 down-regulated differential metabolites were O-feruloyl coumarin, D-erythro-sphinganine, coumarin O-rutinoside, tricin 5-O-hexoside, 3-(4-hydroxyphenyl)propionic acid, eriodictiol C-hexosyl-O-hexoside N-acetyl-L-tyrosine, sakuranetin, hesperetin O-hexosyl-O-hexoside, N-p-coumaroyl hydroxyagmatine.

### 3.6 Analysis of KEGG enrichment pathways for differential metabolites

KEGG pathway enrichment analysis of the differentially expressed metabolites was performed using Metaboanalyst 4.0. The top 20 significantly enriched metabolic pathways are presented in **Figure 4D**, including flavonoid biosynthesis, phenylpropanoid biosynthesis, biosynthesis of phenylpropanoids, ubiguinone and other terpenoid-guinone biosynthesis, longevity regulating pathway, toluene degradation, dopaminergic synapse, stilbenoid, diarylheptanoid and gingerol biosynthesis, asthma, betalain biosynthesis, biosynthesis of enediyne antibiotics, biosynthesis of vancomycin group antibiotics, bisphenol degradation, fc epsilon RI signaling pathway, folate biosynthesis, histamine H2/H3 receptor agonists/antagonists, monoterpenoid biosynthesis, phosphatidylinositol signaling system, PI3K-akt signaling pathway, puromycin biosynthesis.

### 3.7 Integrated analysis of metabolomic and transcriptomic of tomato in the two groups

**Figure 5:**
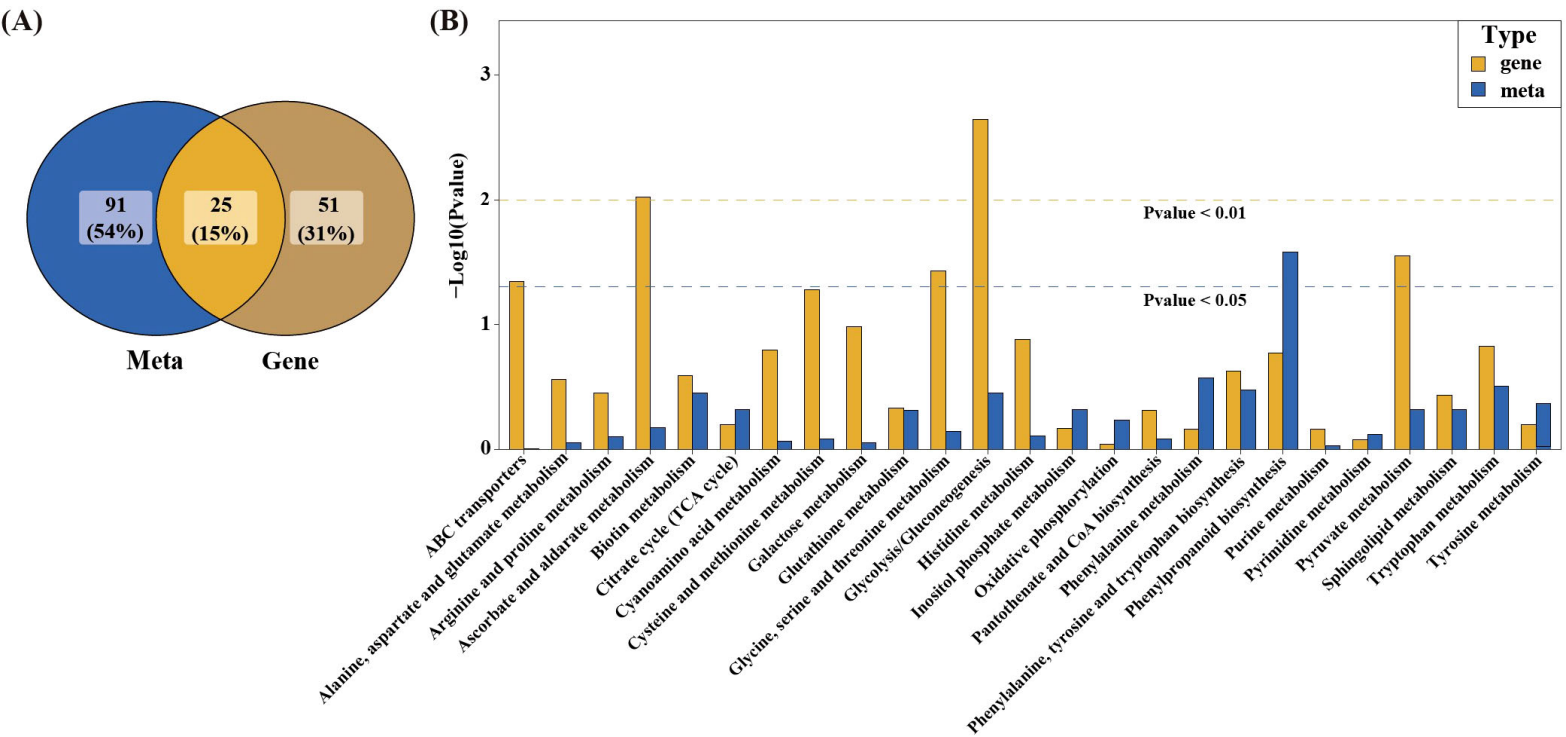
Combined analysis of the metabolic and transcriptional profiles of *SlBEL11*-RNAi tomatoes compared to WT group. **(A)** Venn diagram to show the number of shared KEGG pathways enriched by DEGs (Gene) and DEMs (Meta). **(B)** Bar chart to show the p-values of the enriched KEGG pathways. The x-axis represents the KEGG pathways, and the y-axis indicates the -log_10_p-value. The dashed lines drawn at -log_10_(0.05) marks the statistical significance threshold.

KEGG enrichment analysis of differential genes and metabolites identified 25 co-enriched KEGG-enriched pathways (**Figure 5A**). To further explore the relationship between DEMs and DEGs and determine the pathways affected by SlBEL11, we overlaid p-values thresholds on KEGG histograms, prioritizing pathways enriched by both DEMs (*p* < 0.05) and DEGs (*p* < 0.01) (**Figure 5B**). This approach identified six key pathways, including ABC transporters, ascorbate and aldarate metabolism, glycine/serine/threonine metabolism, glycolysis/gluconeogenesis, phenylpropanoid biosynthesis, and pyruvate metabolism.

**Figure 6:**
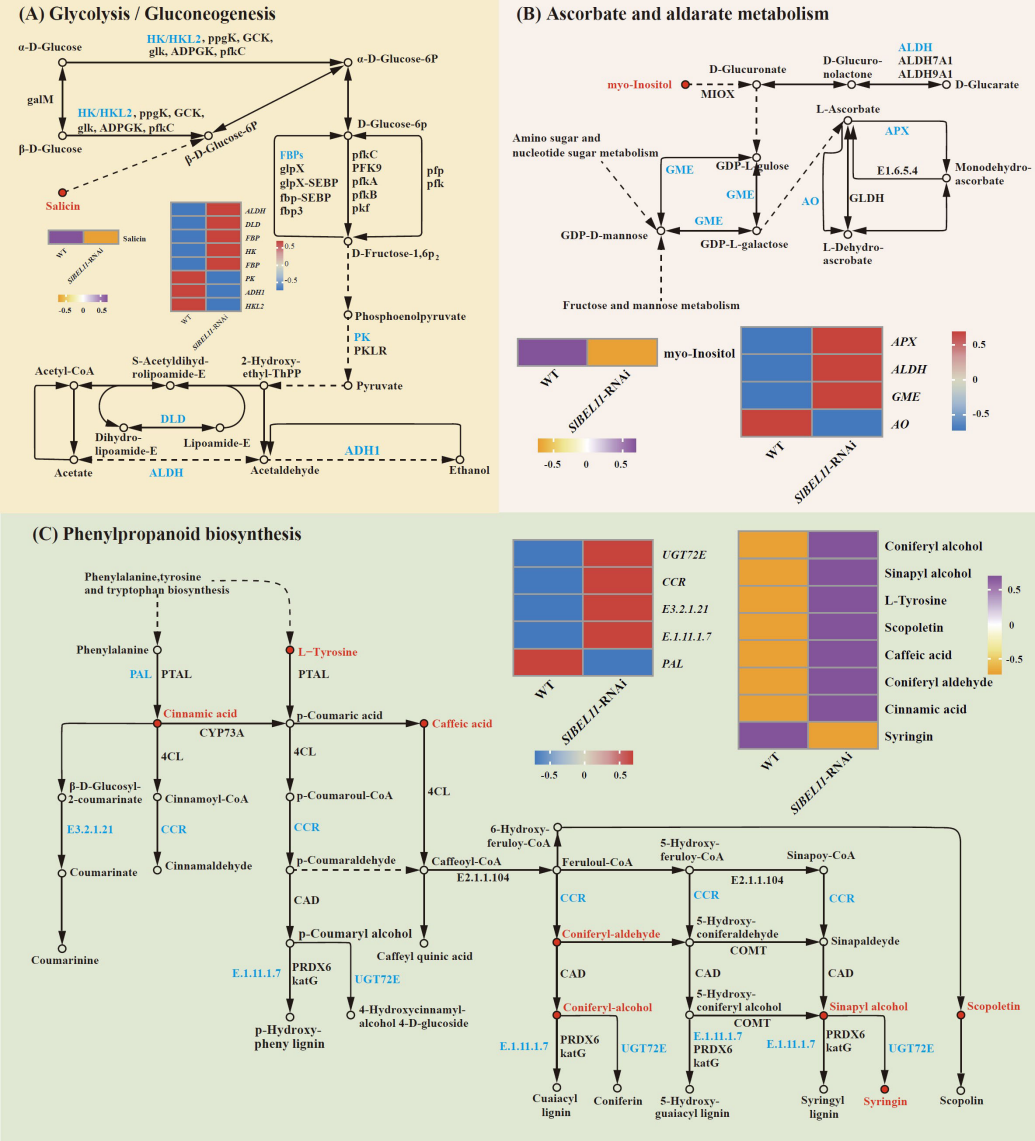
The differential metabolites and differential gene regulatory networks related to SlBEL11 in tomatoes. **(A)** Glycolysis/Gluconeogenesis Pathway. **(B)** Ascorbate and aldarate metabolism. **(C)** Phenylpropanoid biosynthesis. The arrows connecting the metabolites represent genes, and the circular diagrams represent metabolites. Genes in red indicate upregulation, while those in blue indicate downregulation. Metabolites in purple indicate upregulation, and those in orange indicate downregulation.

**Figure 7:**
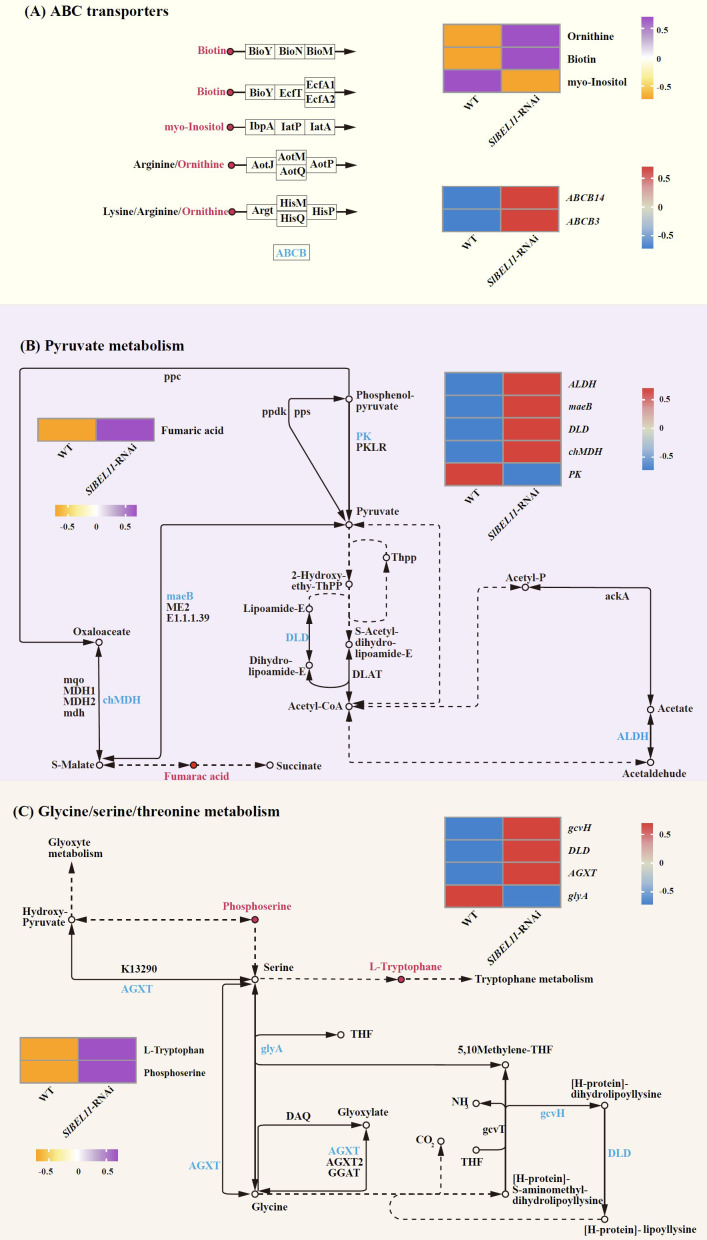
The differential metabolites and differential gene regulatory networks related to SlBEL11 in tomatoes. **(A)** ABC transporters pathway. **(B)** Pyruvate metabolism. **(C)** Cysteine, serine and threonine metabolism. The arrows connecting the metabolites represent genes, and the circular diagrams represent metabolites. Genes in red indicate upregulation, while those in blue indicate downregulation. Metabolites in purple indicate upregulation, and those in orange indicate downregulation.

Expression and regulatory patterns of differential metabolites and genes associated with glycolysis/gluconeogenesis, ascorbate/aldarate metabolism, and phenylpropanoid biosynthesis are summarized in **Figure 6**. In glycolysis/gluconeogenesis, salicin decreased twofold, accompanied by downregulation of *ADH1* (3.3-fold) and *PK* (2.4-fold) (**Figure 6A**, **Supplementary Table S7**). For ascorbate/aldarate metabolism, inositol declined 2.7-fold, while *APX* (24-fold), *ALDH* (5.7-fold), and *GME* (2.1-fold) were upregulated, contrasting with the marked suppression of *AO* (5.7-fold) (**Figure 6B**, **Supplementary Table S7**). In phenylpropanoid biosynthesis, seven metabolites, including coniferyl alcohol (5.1-fold), sinapyl alcohol (4.7-fold), L-tyrosine (4.6-fold), Scopoletin (2.9-fold), caffeate (2.6-fold), coniferyl aldehyde (2.6-fold) and cinnamic acid (2.2-fold), showed elevated abundance, whereas syringin declined 5.9-fold. Concurrently, *UGT72E* (69.2-fold), *CCR* (11.3-fold) and *E1.11.1.7* (2.3-fold) were upregulated, opposing the 1.9-fold downregulation of *PAL* (**Figure 6C**, **Supplementary Table S7**). **Figure 7** illustrates coordinated metabolic and transcriptional interactions across ABC transporters, pyruvate metabolism, and glycine/serine/threonine metabolism. In the category of ABC transporters, ornithine (5.3-fold) and biotin (4.3-fold) accumulated, while inositol decreased 2.7-fold alongside the upregulation of *ABCB14* and *ABCB3* (8-fold and 7.6-fold, respectively) (**Figure 7A**, **Supplementary Table S7**). Pyruvate metabolism exhibited fumaric acid accumulation with four upregulated genes, including *ALDH* (5.7-fold), *maeB* (5.4-fold), *DLD* (4.8-fold) and *chMDH* (2.2-fold), contrasting with the suppression of PK (2.4-fold) (**Figure 7B**, **Supplementary Table S7**). Glycine/serine/threonine metabolism featured elevated L-tryptophan (4.0-fold) and phosphoserine (2.2-fold), concurrent with upregulation of *gcvH*(5.4-fold), *DLD* (4.8-fold) and *AGXT* (2.9-fold), opposing the downregulation of *glyA* (2.2-fold). (**Figure 7C**, **Supplementary Table S7**).

## 4 Discussion

The transcription factor SlBEL11, a member of the BEL1-like family, has emerged as a key regulator of plant development in recent studies (Meng et al., 2018; He et al., 2022a; Dong et al., 2024). Our integrated multi-omics approach unveiled its comprehensive influence on transcriptional reprogramming and metabolic remodeling across six interconnected pathways, providing mechanistic insights into its role in coordinating ripening-associated physiological transitions.

Ascorbic acid (vitamin C), a critical antioxidant in fruits, governs ripening and postharvest storage quality through its dynamic accumulation (Corpas et al., 2024; Lin et al., 2025). The ascorbate metabolism pathway serves as a critical node in SlBEL11-mediated regulation. In *SlBEL11*-RNAi fruits, despite significant downregulation of L-galactose pathway rate-limiting enzyme *GME* (2.1-fold upregulation), which typically drives ascorbate biosynthesis (Zheng et al., 2022), we observed depleted myo-inositol levels (2.7-fold decrease) (**Figure 6B**, **Supplementary Table S7**). This paradox suggests preferential metabolic flux diversion through the alternative L-gulose salvage pathway, likely compensating for restricted precursor availability. Simultaneous suppression of ascorbate oxidase (*AO*, 5.7-fold) aligns with elevated *APX* (24-fold) and *ALDH* (5.7-fold) expression, indicating a strategic trade-off between ascorbate degradation inhibition and enhanced antioxidant capacity (**Figure 6B**, **Supplementary Table S7**). Such coordinated regulation ensures sufficient hydroxyproline biosynthesis for cell wall cross-linking while mitigating oxidative stress—a dual mechanism underlying the observed 30% firmness reduction (Wu et al., 2024). Notably, this metabolic tension mirrors findings in *SlBL4*-mutant tomatoes (Yan et al., 2020), suggesting a conserved BEL-family regulatory paradigm in redox-structural coupling.

The phenylpropanoid pathway constitutes a central metabolic network in plant secondary metabolism, respobsible for the biosynthesis of lignin, flavonoid derivatives, and phenolic acid compounds that collectively mediate cell wall reinforcement and oxidative stress mitigation (Anwar et al., 2021; Yao et al., 2021; Liang et al., 2024). Which displayed hierarchical dysregulation characterized by upstream repression and terminal activation. While *PAL* suppression (1.9-fold) constrained cinnamic acid biosynthesis, consequent accumulation of L-tyrosine (4.6-fold) and cinnamic acid (2.2-fold) implies alternative substrate provisioning through tyrosine ammonia-lyase (TAL) activity—a compensatory mechanism previously undocumented in BEL-regulated systems (**Figure 6C**, **Supplementary Table S7**). Downstream activation of *CCR* (11.3-fold) and *UGT72E* (69.2-fold) contrasts sharply with syringin depletion (5.9-fold), revealing metabolic bottlenecks at monolignol glycosylation steps (**Figure 6C**, **Supplementary Table S7**). This transcriptional-metabolic disconnect may arise from substrate competition between UGT72E isoforms, as evidenced by differential affinity for coniferyl/sinapyl alcohol derivatives (Anwar et al., 2021). The net physiological outcome—reduced lignification coupled with enhanced soluble phenolic accumulation—mirrors the “metabolic channeling” strategy observed in pathogen-challenged plants (Yao et al., 2021), positioning SlBEL11 as a plasticity regulator during ripening-stress cross-talk.

As the central energy-converting hub of sugar metabolism, the glycolysis/gluconeogenesis pathway underpins cellular energy supply during fruit ripening (Stroka et al., 2024). *SlBEL11* knockdown induced a paradoxical glycolytic profile: upregulated *HK* and *ADH1* contrasted with *PK* suppression and salicin depletion (**Figure 6A**, **Supplementary Table S7**). This pattern suggests bifurcated carbon flux—enhanced sucrose cleavage drives ethanolic fermentation rather than mitochondrial respiration, potentially optimizing ATP yield under reduced TCA cycle activity. The resultant NAD+ regeneration could mitigate ROS accumulation from RBOH-mediated respiratory burst (Jones et al., 2007), explaining maintained fruit integrity despite accelerated softening. Such metabolic flexibility aligns with the “overflow hypothesis” in glycolytic regulation (Liu et al., 2021), establishing SlBEL11 as an energy rheostat balancing catabolic efficiency and oxidative damage.

The ABC transporter system emerged as a SlBEL11-dependent hub for secondary metabolite trafficking. While *ABCB14* (8-fold) and *ABCB3* (7.6-fold) induction typically enhances phytoalexin efflux (Gani et al., 2021), concomitant myo-inositol depletion suggests compromised osmoregulation-mediated turgor maintenance (**Figure 7A**, **Supplementary Table S7**). This creates a metabolic dilemma—increased defense compound export vs. cellular dehydration risk. The ornithine/biotin accumulation-inositol depletion axis mirrors stress-adapted solute redistribution in drought-tolerant cultivars (Liang et al., 2024), implying SlBEL11’s role in abiotic-biotic stress integration during ripening.

## 5 Conclusion

This study unveils the mechanism by which the transcription factor SlBEL11 regulates in tomato fruit ripening. Through integrated transcriptomics and metabolomics analyses, we demonstrate that SlBEL11 modulates gene expression and metabolite accumulation across critical pathways, including ABC transporters, ascorbate and aldarate metabolism, glycine/serine/threonine metabolism, glycolysis/gluconeogenesis, phenylpropanoid biosynthesis, and pyruvate metabolism. These pathways collectively govern fruit nutritional quality, firmness, antioxidant capacity and ripening initiation. SlBEL11 affects ascorbate homeostasis and cell wall remodeling by regulating ascorbic acid metabolism, enhances phenolic compounds accumulation and antioxidant defenses via phenylpropane pathway activation, fine-tunes energy metabolism through modulation of sugar catabolism, with downstream impacts on redox homeostasis. Meanwhile, SlBEL11 influences the ABC transporter-mediated pathway to alter the transmembrane transport of secondary metabolite trafficking and boosts pathogen defense mechanism. Collectively, our findings reveal a multi-layered regulatory network through which SlBEL11 integrates metabolic, structural, and defensive processes during fruit ripening.

## Data Availability

The data that support the findings of this study are openly available in the National Center for Biotechnology Information (NCBI) SRA database under the BioProject ID: PRJNA1301375.

## Supplementary material

Supplementary Figure 1 Quality control of RNA-Seq sequencing for two groups of tomatoes.

Supplementary Figure 2 Transcriptome validation analysis of RNA-seq data quality metrics and RT-qPCR verification of DEGs in WT vs. *SlBEL11*-RNAi fruits at the breaker (Br) stage.

Supplementary Figure 3 Metabolic profile quality control. Total ion current (TIC) chromatograms of QC samples from tomato fruit metabolomics.

Supplementary Figure 4 Metabolite data reliability assessment. Correlation clustering heatmap of QC samples.

Supplementary Figure 5 Hierarchical clustering analysis of differential metabolites. Heatmap displaying normalized abundance profiles.
