# Supplementary material for: Integrated transcriptomic and metabolomic analyses elucidate the regulatory role of SlBEL11 in tomato fruit ripening
Source: Front Plant Sci. 2025 Sep 2;16:1666515. doi: 10.3389/fpls.2025.1666515 (PMC12436317; doi:10.3389/fpls.2025.1666515)
Supplement: Supplementary Figure 1 — Quality control of RNA-Seq sequencing for two groups of tomatoes. [file DataSheet1.pdf]

# Supplementary figures

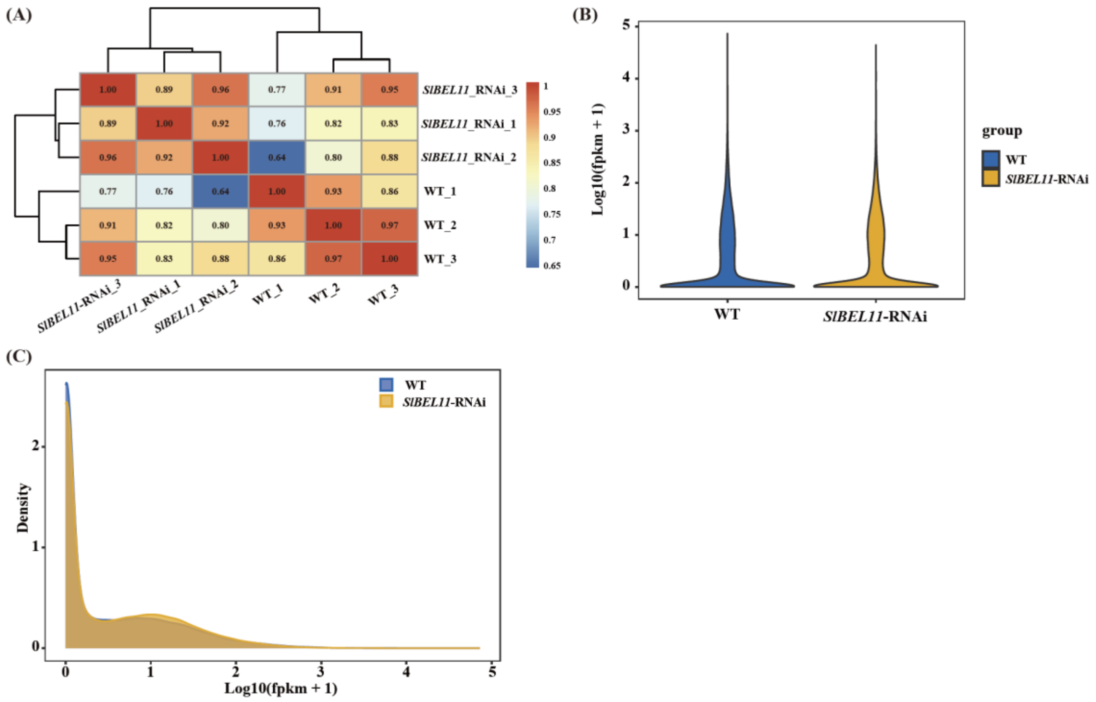


**Figure S1.** Quality control of RNA-Seq sequencing for two groups of tomatoes. (A) Correlation clustering heatmap analysis of WT and *SlBEL11*-RNAi RNA samples. (B) Violin plot of gene expression levels normalized in FPKM value. (C) Gene expression density distribution map.

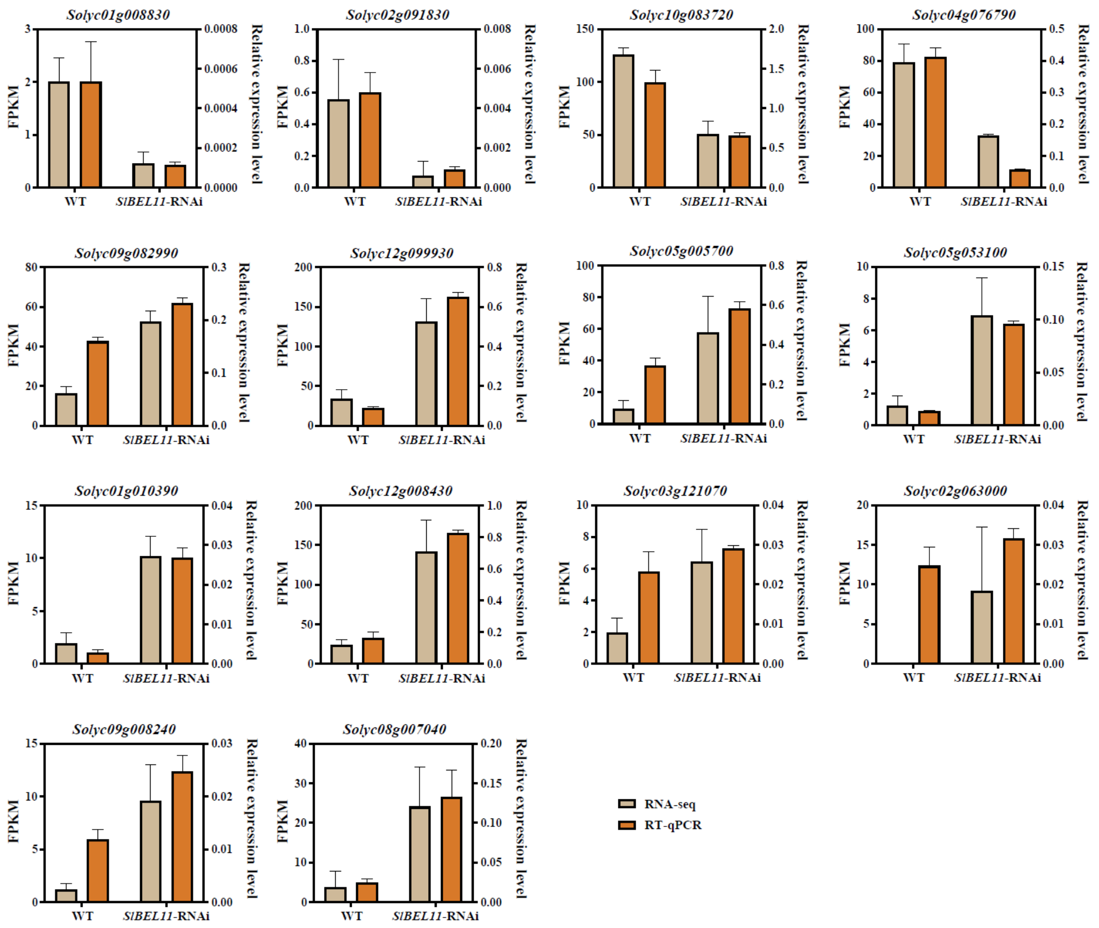


**Figure S2.** RNA-seq analysis and quantitative RT-qPCR validation of differentially expressed genes between Wild-Type and SlBEL11-RNAi in tomato fruit during the Br stage. Actin gene was used as the reference gene. Technical replicates were performed in triplicate.

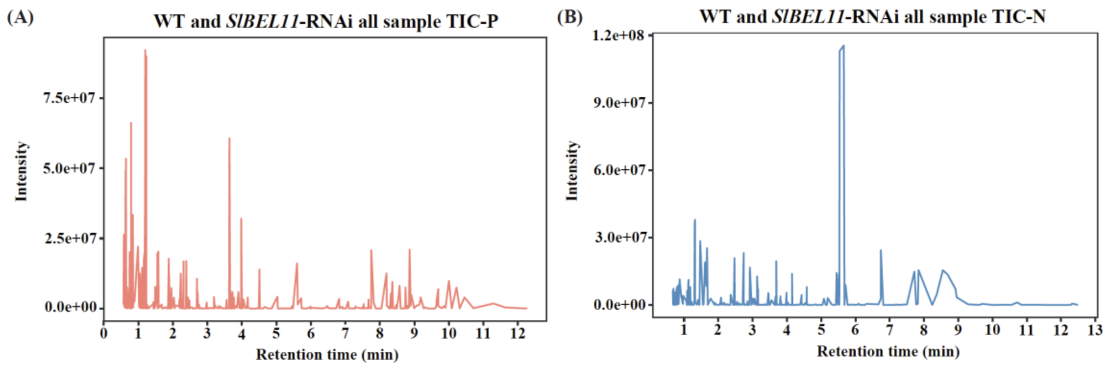


**Figure S3** Total ion current chromatogram of the QC samples in tomato. (A) TIC of the sample in positive ion mode. (B) TIC of the sample in negative ion mode.

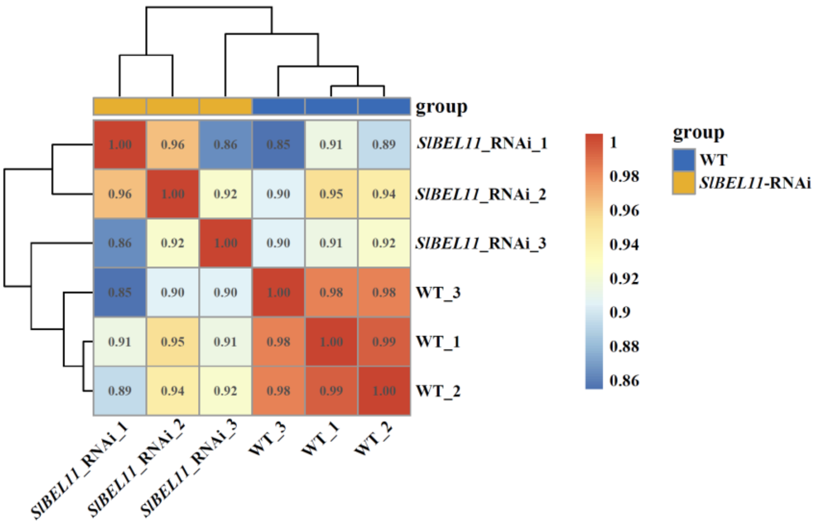


**Figure S4** Correlation Clustering Heatmap of QC samples of metabolome.

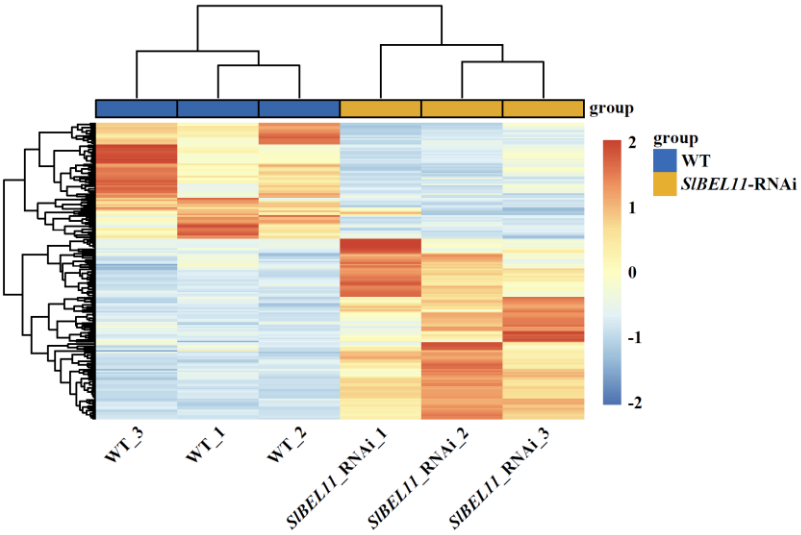


**Figure S5** Heatmap of clustered differential metabolites.
